# Expert consensus on designing a metaverse supported blended EFL module in chinese higher education: A Fuzzy Delphi method

**DOI:** 10.1371/journal.pone.0347027

**Published:** 2026-04-16

**Authors:** Yuliang Jiao, Dorothy DeWitt, Rafiza Abdul Razak

**Affiliations:** 1 School of Foreign Languages, Chongqing Sanxia University of Science and Technology, Chongqing, China; 2 Faculty of Education, University of Malaya, Kuala Lumpur, Malaysia; Kalasalingam Academy of Research and Education, INDIA

## Abstract

Metaverse technologies can provide immersive, interaction-rich experiences for English as a Foreign Language (EFL) learning, yet curriculum-level design principles and implementation guidance remain limited. This study developed, through expert consensus, a ranked blueprint for a metaverse-supported blended learning module for undergraduate EFL learners in Chinese higher education. Using an exploratory design, Phase 1 elicited candidate elements through semi-structured interviews with EFL instructors (n = 5), and Phase 2 applied the Fuzzy Delphi Method (FDM) with a 12-expert panel using a five-point Likert scale. Items were retained only if they met all prespecified criteria (Agreement ≥ 75%, interquartile range [IQR] ≤ 1.0, and fuzzy distance [d] ≤ 0.20). Retained items were prioritized by defuzzified value (DV), with ties resolved by IQR, then d, then Agreement. Actionable consensus was reached across six domains: learning objectives, learning content, instructional strategies, learning activities, assessment methods, and learning resources. Resampling-based stability checks (leave-one-out and bootstrap) supported the robustness of the induced priority ordering. Kendall’s *W* indicated limited overall concordance across heterogeneous items; accordingly, item inclusion relied on the prespecified thresholds. The study contributes a replicable, ranked blueprint that embeds constructive alignment in immersive EFL contexts and provides an implementation-ready specification to support staged adoption and subsequent validation in higher education.

## 1. Introduction

Competency in English as a Foreign Language (EFL) is important as it can help develop skills such as communication and critical thinking to prepare learners for life in the 21^st^ century, and to remain competitive globally in an increasingly interconnected world [1]. Hence, in higher education institutions, EFL courses are increasingly expected to cultivate communicative competence rather than rote knowledge. However, conventional lecture centered practices often underserve authentic language use and learner engagement, thus motivating the call to realign intended outcomes, tasks, and assessments through constructive alignment and backward design [2,3]. Blended learning has the potential to enhance flexibility and interaction, especially when pedagogy, not technology, drives the design choices [4,5]. In this sense, conventional EFL typically centers on classroom explanation and discrete exercises, whereas metaverse supported blended approaches deliberately integrate immersive tasks with face-to-face scaffolding and online components to serve explicit language outcomes.

In this study, these ideas are operationalized in a metaverse supported blended EFL module, where metaverse activities are integrated with classroom and online components at the module level. Recent advances in metaverse have extended blended learning by enabling situated, experiential tasks and heightened social presence. The higher education literature suggests that the Metaverse can support contextualized practice when instructional goals, scaffolding, and feedback are explicitly specified [6]. For EFL specifically, communicative and task based paradigms offer a natural anchor as immersion and presence alone are insufficient, unless activities require goal directed interaction and produce assessable artefacts [7–9]. Thus, the key contrast with conventional lecture centered EFL is not technology per se, but whether instructional design reliably elicits meaning focused interaction and observable performance evidence. Likewise, game elements should be designed for learning rather than adopted for novelty, otherwise their effect on learning outcomes is likely to be limited [10].

Despite these developments, there is still limited curriculum level guidance for designing modules for metaverse supported EFL, particularly in higher education systems. Further, research in this area remains scattered. Many prior studies focus on learner perceptions, prototype platforms, or learning outcomes [11–13], but they seldom offer a consensus based, ranked set of design elements that curriculum teams can readily adopt and adapt. This gap is especially consequential in large systems such as Chinese higher education, where the scale and diversity of implementation necessitate clearly articulated priorities across objectives, strategies, activities, assessments, and resources that can accommodate different bandwidth and device constraints as well as varied levels of teacher readiness and institutional support.

The present study addresses this gap by developing an expert validated, ranked blueprint for a metaverse supported blended EFL module in higher education. It adopts an exploratory sequential design in which qualitative insights from EFL instructors are first used to identify candidate design elements, and these elements are then refined and prioritized through a Fuzzy Delphi consensus process with experts, using strict thresholds to screen and rank the items. In doing so, the study seeks to clarify which module components should be considered essential and how experts prioritize them for implementation.

Against this backdrop, the study investigates the following research questions:

1. Which elements across objectives, content, strategies, activities, assessments, and resources achieve consensus among experts for inclusion in a metaverse supported EFL blended module for higher education?
2. How do experts prioritize these elements in terms of their importance for the adoption of a metaverse supported blended EFL module in higher education?

By reporting both consensus and ranking of agreement according to priority, the study moves beyond a simple list of features, to provide a replicable decision-making tool for curriculum developers. The resulting blueprint aligns communicative outcomes with immersive tasks and assessment evidence, offering the principles for the rollout of metaverse supported blended EFL module for higher education. The findings can guide educators and curriculum developers in designing innovative EFL modules for the digital age.

## 2. Literature review

### 2.1. Theoretical framework

The metaverse is commonly described as a persistent, shared, three-dimensional digital space where users, via avatars, interact with other agents and objects in real time, typically enabled through immersive technologies such as virtual and augmented reality (VR/AR). In education, metaverse environments promise movement from content transmission to enactive, social, and situated learning, aligning with constructivist views that knowledge is actively built through purposeful activity and interaction [14,15]. Within situated cognition, authentic contexts and social participation are central; by recreating context-rich scenarios, metaverse spaces allow learners to rehearse communicative functions that mirror real-world exchanges [11]. In EFL settings, such environments can embed linguistic input, interaction, and cultural content within coherent scenarios rather than decontextualized drills, thereby supporting both language development and digital literacy.

A blended learning stance integrates in person and online elements to combine flexibility with interaction [4]. When blended designs embed metaverse components, instructors can orchestrate extended reality tasks that link face to face scaffolding with immersive practice, potentially mitigating limits of conventional online learning while maintaining structure and access [16,17]. From a curriculum perspective, metaverse activities thus function as one strand within a broader blended module, where classroom, online, and immersive components must be planned as an integrated whole rather than as isolated add-ons.

Curriculum alignment in such settings benefits from three complementary frameworks. Firstly, constructive alignment emphasizes coherence among intended learning outcomes, learning activities, and assessment [2]; in metaverse-based EFL, this implies mapping language functions and skills to task designs and evidence of performance. Secondly, the Community of Inquiry (CoI) model focuses on cognitive, social, and teaching presence as mutually reinforcing conditions for deep learning [4]. The affordances of metaverse are that it can intensify social presence (co-location, co-action) while interactive tasks and facilitation strategies sustain teaching and cognitive presence. Thirdly, TPACK conceptualizes teachers’ integrated technological, pedagogical, and content knowledge [18]. In practice, successful metaverse modules hinge on teachers’ ability to fuse language-pedagogical intentions with immersive tools, for example, designing avatar mediated role plays that target functional language and interactional competence. These frameworks highlight that teachers must be ready not only to operate metaverse platforms but also to design aligned tasks and assessments, an issue that is particularly salient in large EFL systems such as Chinese higher education. This view aligns with work on embedding technology in curriculum design, which argues that digital tools should be integrated at the level of learning outcomes, activities, and assessment rather than added as standalone features, while also considering cost, appropriateness, and teacher training [19].

Together, these lenses suggest that metaverse adoption should not be driven by novelty. Rather, tasks, resources, and assessments must be explicitly aligned to language outcomes and embedded within a social learning design that instructors can reliably enact. What remains underspecified in the literature is how such aligned designs can be articulated at the module level in the form of concrete, prioritized components that curriculum teams can implement.

### 2.2. Prior research on metaverse in EFL

Recent EFL studies have moved beyond proof of concept to probe skill specific gains, motivational mechanisms, and design contingencies. A consistent pattern is that metaverse/XR benefits surface when tasks are authentic, dialogic, and goal directed, and when instructional orchestration manages cognitive load and supports feedback cycles. At the same time, reported effects are not uniformly positive across studies, and variations in task design, duration, measurement choices, and implementation conditions suggest that “immersion” is a necessary but insufficient condition for learning gains.

On productive skills, immersive experiences have been linked to improved speaking performance and confidence. In higher education EFL, Hwang and Lee reported gains in oral proficiency when learners practiced speeches and drama via avatars, arguing that presence and reduced anxiety mediated improvement [20]. A related comparison indicated that 3D metaverse platforms fostered higher engagement and intention to use than 2D environments, highlighting presence as a motivational driver [21]. On pronunciation and fluency, VR studies that embed situated interaction, for example, service encounters and guided tours, show improvements when feedback and rehearsal are built into the cycle [16]. These findings imply that affective mechanisms, for example, reduced anxiety and increased willingness to communicate, may operate as pathways, but their instructional value depends on whether interaction is structured toward assessable communicative outcomes.

Evidence on lexical and mixed skills outcomes is also positive when tasks integrate elaboration and retrieval within meaningful activity. Reviews of XR in education and language learning note stronger effects where assessment and teacher facilitation are specified and where connectivity and cognitive load are managed; otherwise, benefits attenuate [6,16]. Complementing outcomes research, perception studies generally find favorable student attitudes toward immersive EFL, tempered by concerns about technical reliability and instructional clarity. For example, Rojas et al. documented enthusiasm for immersion yet mixed views on its impact when learning goals and assessments were underspecified [22]. However, most of these studies remain tool or activity centered, offering limited guidance on how to structure an entire metaverse-supported EFL module with clearly sequenced objectives, activities, and assessments.

Across this literature, two design regularities recur: (a) immersion adds value when it lowers affective barriers and enables meaningful interaction, not when it merely relocates lectures into 3D; and (b) untethered gamification and content not mapped to language outcomes tend to yield weaker effects [16]. These findings reinforce the need for module-level blueprints that align outcomes, tasks, assessment, and resources in ways that are both pedagogically principled and operationalizable. In addition, implementation feasibility increasingly depends on learners’ and teachers’ digital literacies and on institutional safeguards, suggesting that design guidance should integrate pedagogical alignment with contextual adaptability.

### 2.3. Methodological benchmarks for the FDM in education

For domains with emergent evidence, FDM provide a structured procedure to elicit and stabilize expert judgements. Best practice guidance emphasizes (i) transparent panel eligibility and diversity, (ii) a priori definitions of consensus, and (iii) explicit stopping and stability rules [23,24]. Reviews of FDM studies show that a 75% agreement is commonly used yet not always prespecified; stronger studies therefore articulate parallel criteria rather than relying on a single indicator [25,26]. Importantly, when the goal is design specification, that is, screening and prioritizing candidate elements, Delphi outputs are best interpreted as structured expert judgment evidence rather than causal effectiveness evidence.

FDM extends the classical Delphi method by mapping linguistic ratings to triangular fuzzy numbers (TFNs), aggregating expert TFNs, and defuzzifying to obtain crisp priority values [27]. In education and adjacent design contexts, many FDM applications adopt convergent retention gates, such as a fuzzy distance threshold around d ≤ 0.20 together with ≥75% agreement on item importance; some studies additionally report an α-cut/defuzzified value (e.g., DV ≥ 0.50) for retention or prioritization [27,28]. Narrative reviews also note that fuzzy variants can reduce the rounds to reach stable consensus, as fuzzy aggregation captures nuance in expert ratings more efficiently than crisp scales. In the present study, three parallel criteria: Agreement ≥ 75%, IQR ≤ 1.0, and d ≤ 0.20, were specified; and DV was strictly for ranking accepted items, thereby avoiding overreliance on any single metric. Accordingly, “consensus” in this study refers to threshold-based endorsement and prioritization of design elements, not to convergence demonstrated through repeated feedback cycles.

To examine panel level stability of the induced rankings, we computed Kendall’s coefficient of concordance (*W*); values around 0.10 ~ 0.30 typically indicate low concordance, 0.50 ~ 0.70 moderate concordance, and ≥0.70 strong concordance [29–31]. Together, transparent sampling, a priori thresholds, item level retention rules, and stability checks (IQR, Kendall’s *W*) comprise a defensible benchmark when the goal is to deliver prioritized, implementation ready design specifications rather than broad narrative recommendations. In multi domain item sets, Kendall’s *W* should be interpreted as an overall concordance indicator across heterogeneous items, while item level retention thresholds provide the primary acceptance evidence.

### 2.4. Research gap and consolidation to research questions

Despite accelerating interest, most metaverse EFL publications remain tool or task centric and do not culminate in alignment ready, ranked blueprints for sustained delivery in university EFL programs. Prior frameworks summarize affordances and challenges but rarely (a) prioritize what to include first under real constraints (devices, bandwidth, teacher readiness), or (b) map outcomes to strategies, activities, assessment, and resources at module level [4,16]. Methodologically, few studies adopt FDM with explicit, parallel thresholds plus stability checks (IQR; Kendall’s *W*) to produce a replicable, ranked specification [23,26,28]. Evidence grounded in Chinese higher education, the world’s largest EFL context, remains scarce in terms of module design consensus and prioritization. By delivering a ranked, consensus-based design specification across these domains, this study provides curriculum teams with actionable priorities to support staged adoption under institutional constraints and to inform subsequent evaluation work in metaverse enhanced EFL.

## 3. Methodology

### 3.1. Research design

This study employed an exploratory sequential mixed methods design to develop a metaverse supported blended learning module for undergraduate EFL courses in China. Phase 1 elicited qualitative insights via semi-structured interviews with five experts in EFL pedagogy, curriculum design, and educational technology to generate a theoretically and practically grounded item pool. Phase 2 applied a single round FDM with a 5 point Likert scale to quantify consensus among a broader expert panel (n = 12) and to refine the proposed elements.

A single round was adopted to minimize panel burden and avoid attrition after a rigorous item generation phase. In line with methodological guidance on FDM in education and design, fuzzy aggregation was used to capture nuanced expert judgements while allowing consensus to be reached within one iteration [23,28]. We therefore prespecified parallel, item level consensus gates—percent agreement, IQR, and fuzzy distance *d*, and used the defuzzified value (DV) for prioritizing accepted items rather than as an additional retention threshold; Kendall’s *W* was reported as a panel level concordance index. Accordingly, consensus in this study refers to threshold-based endorsement and prioritization of design elements within a single round FDM procedure, and does not constitute evidence of post-implementation effectiveness. This design balances qualitative depth with quantitative rigor and enhances transparency and reproducibility for emerging metaverse based curriculum work.

### 3.2. Participants

Two expert cohorts contributed, with partial overlap for continuity.

**Phase 1 (Interviews)**. Five experts met the following criteria: (a) demonstrated expertise in EFL pedagogy and/or curriculum or educational technology design; (b) at least 8 years of relevant experience; and (c) recent involvement in technology enhanced language teaching or course design. Interviews were conducted in English or Chinese according to participant preference.

**Phase 2 (FDM panel)**. A maximum variation sample of 12 experts (7 female; 5 male) was assembled to represent diverse ranks (lecturer, associate or full professor) and domains (EFL instruction, instructional design, educational technology, English linguistics, educational psychology) [32]. Inclusion required either a doctoral degree or an associate professorship and at least 8 years of directly relevant experience. This composition provided disciplinary breadth for judging objectives, content, strategies, activities, assessments, and resources. This panel size is consistent with methodological recommendations for FDM studies, which commonly suggest panels of around 10–18 carefully selected experts when the goal is to elicit structured expert judgment rather than to estimate population parameters [24,33]. Table 1 summarizes panel characteristics (interview experts correspond to IDs 1, 6, 7, 8, and 10).

**Table 1:** Panel of experts for the FDM questionnaire (n = 12).

No.	Gender	Experience (Years)	Level of Education	Position	Expertise				
					TEFL	Curriculum & Instruction	Educational Technology	English Linguistics	Educational Psychology
1	Male	13	PhD	Professor	√	√			
2	Female	12	PhD	Associate professor	√		√		
3	Male	10	PhD	Lecturer		√		√	
4	Male	13	Master	Associate Professor		√	√		
5	Female	17	PhD	Associate professor	√		√		
6	Male	11	PhD	Lecturer	√			√	
7	Female	14	PhD	Associate Professor	√	√		√	
8	Male	15	PhD	Associate Professor	√			√	
9	Female	8	PhD	Lecturer			√	√	
10	Female	18	PhD	Professor	√		√		
11	Female	9	PhD	Associate Professor					√
12	Female	8	Master	Associate Professor					√

*Note*. Inclusion required doctoral degree or an associate professorship and 8–18 years relevant experience. Interview experts: Nos. 1, 6, 7, 8, 10.

**Participants, ethics, and consent.** A total of 12 adult experts were recruited between 10 March 2024 and 25 March 2024. Written informed consent was obtained electronically via *Wechat* prior to data collection. Only professional background variables were collected; no personally identifiable information was recorded. Written informed consent was obtained, and all responses were anonymized before analysis. Ethical approval was obtained from the Ethics Committee of the University of Malaya (UM.TNC2/UMREC_2883). No minors or vulnerable populations were involved.

### 3.3. Instruments

**Interview protocol**. Guided by curriculum design frameworks [3], the bilingual (English–Chinese) protocol covered six domains: learning objectives, content, instructional strategies, learning activities, assessment, and resources. Two bilingual educators reviewed the protocol for clarity and cultural appropriateness; minor wording adjustments followed. One to one interview (in person or online about 30–50 minutes) were audio-recorded with consent. The first author conducted all interviews using non-leading probes.

**Fuzzy Delphi questionnaire**. Items generated from Phase 1 thematic analysis were organized under the six domains above. Panelists rated essentiality on a 5-point Likert scale (1 = strongly disagree, to 5 = strongly agree) and could provide open comments or propose new items. A small pilot with two EFL instructors (not on the panel) led to minor refinements in wording/layout.

### 3.4. Data collection and analysis

(1) Phase 1: Qualitative Interviews

Interviews moved from broad views of the metaverse in EFL to concrete module components. Recordings were transcribed verbatim and analyzed thematically. Two researchers independently coded excerpts within the six a priori domains while allowing emergent codes; discrepancies were resolved by discussion. Member-checking (participants verified interpretive summaries) and peer debriefing enhanced trustworthiness. The analysis produced the initial item pool for the FDM.

(2) Phase 2: Fuzzy Delphi questionnaire (5-point scale)

The questionnaire was administered electronically and completed anonymously. Researchers acted as neutral facilitators clarifying instructions without influencing judgments. Given the extensive item generation stage, a single round FDM was implemented with strict consensus thresholds and stability diagnostics to reach a defensible level of agreement within one iteration.

(3) FDM computation

**Step 1. Fuzzification (linguistic rating to TFN).**

Each 5-point Likert rating (1 ~ 5) was mapped to a triangular fuzzy number (TFN) on [0,1] using an established linguistic term set (Table 2). For example, a rating of 5 (“strongly agree”) corresponds to the TFN (0.6,0.8,1.0). This fuzzification captures the uncertainty inherent in expert judgments and aligns with prior FDM practice in education and design research [23,28,34].

**Table 2:** Linguistic variables and corresponding fuzzy values.

Likert scale	Linguistic variable	Fuzzy scale
1	Strongly disagree	(0.0, 0.0, 0.2)
2	Disagree	(0.0, 0.2, 0.4)
3	Neutral	(0.2, 0.4, 0.6)
4	Agree	(0.4, 0.6, 0.8)
5	Strongly agree	(0.6, 0.8, 1.0)

*Note.* Fuzzy value mappings adapted from [28].

**Step 2. Aggregation (item-level TFN).**

For each item, expert level TFNs were aggregated component-wise across the 12 experts to obtain the item’s TFN (*m*_1_, *m*_2_, *m*_3_), where *m*_1_, *m*_2_, and *m*_3_ denote the lower, modal, and upper bounds of the panel’s collective judgment.

**Step 3. Defuzzification (centroid)**

The item TFN was converted into a defuzzified value (DV) using the centroid:


$$DV\;=(m_{\text{1}}\;+\;m_{\text{2}}\;+\;m_{\text{3}})/\text{3}\in [\text{0},\text{1}],$$

which reflects the central tendency of expert endorsement for that item. This score reflects the weighted central tendency of expert opinions on each item’s importance. A higher DV (closer to 1) indicates stronger collective endorsement of the item.

**Step 4. Fuzzy distance *d* (dispersion around the panel center).**

Opinion dispersion was quantified by the vertex-wise absolute-average distance between each expert’s TFN and the item’s aggregated TFN, averaged over experts:


$$d=\frac{\text{1}}{n}\sum_{i=\text{1}}^{n}\frac{\left|m_{\text{1}i}-m_{\text{1}}\right|+\left|m_{\text{2}i}-m_{\text{2}}\right|+\left|m_{\text{3}i}-m_{\text{3}}\right|}{\text{3}},\;with\;n=\text{1}\text{2}$$

Smaller *d* values indicate tighter clustering, which indicates stronger consensus.

**Step 5. Item-level dispersion (IQR) and agreement**

Using the raw 5-point ratings, we computed each item’s interquartile range as IQR = *Q*3 − *Q*1, where *Q*1 and *Q*3 are the 25th and 75th percentiles, respectively (software default: inclusive definition). A smaller IQR indicates tighter clustering of expert opinions. In parallel, percent agreement was calculated as the proportion of panelists selecting 4 or 5 on the Likert scale (i.e., endorsing the item as essential). For transparency, we also report the count of raters meeting this threshold (e.g., ≥ 9/12). Missing responses (none in our data; if any) would be handled per item, so that an expert’s missing rating on one item would not eliminate their ratings on other items.

**Step 6. Acceptance rule**

An item was deemed to have reached consensus and was accepted only if it simultaneously satisfied all a priori thresholds: (a) Agreement ≥ 75% (at least 9 of 12 experts rated the item 4 or 5), (b) IQR ≤ 1.0 (on the 5-point scale), and (c) fuzzy distance *d* ≤ 0.20 (representing dispersion around the panel-aggregated triangular fuzzy number). Items failing any threshold were excluded from the final specification (for example, IQR > 1.0: divergent views on feasibility). Domain level acceptance rates were calculated and reported as the number of accepted items divided by the number proposed within each domain. To assess robustness, we conducted sensitivity analyses using nearby cutoffs (IQR 0.8 and 1.2; d 0.15 and 0.25). Acceptance decisions and domain level patterns remained substantively unchanged.

Boundary cases. When an item lay exactly on a boundary (for example, IQR = 1.0 or d = 0.20), it was treated as meeting the corresponding threshold. For transparency, such boundary items were flagged for potential wording clarification in subsequent iterations informed by panel comments.

**Step 7. Ranking and prioritization**

All accepted items were ranked by the defuzzified value (DV) within each domain to indicate relative implementation priority. When DVs were equal, ties were resolved deterministically in the following order: smaller IQR, smaller d, and higher Agreement. For practice-oriented reporting, we provide a Top *k* list for each domain (for example, Top 5) and use these ranked items to build a module blueprint linking objectives to strategies, activities, resources, and assessment.

**(4) Panel level concordance and ranking stability**

To evaluate the stability of the DV based priority ordering under the single round design, we conducted two resampling based robustness checks and treated them as the primary evidence for ranking stability. In the leave one out (LOO) procedure, we removed one expert at a time (12 iterations), recomputed within domain DV rankings, and calculated Spearman’s rank correlation (ρ) between each LOO ranking and the corresponding full sample ranking over retained items. We report the mean ρ and the empirical range. In bootstrap resampling, we sampled experts with replacement (B = 5,000), recomputed within domain rankings, and calculated Spearman’s ρ between each bootstrap ranking and the corresponding full sample ranking. We report the mean ρ and the percentile based 95% interval.

As a supplementary descriptor of overall coordination across heterogeneous items spanning multiple domains, we computed Kendall’s coefficient of concordance (*W*). *W* value ranges from 0 (no agreement) to 1 (complete agreement). Statistical significance was assessed using the chi square approximation, χ² ≈ k(m − 1)*W* (df = m − 1), with a standard tie correction applied when ties occurred.

## 4. Findings

Phase 1 generated a theory and practice informed item pool across six domains, and Phase 2 applied the FDM using a 5-point Likert scale to quantify expert consensus. Across domains, the prespecified criteria yielded consensus, with the following domain-level acceptance rates: learning objectives 7/9 (77.8%), learning content 7/9 (77.8%), instructional strategies 6/10 (60.0%), learning activities 8/9 (88.9%), assessment methods 5/8 (62.5%), and learning resources 6/9 (66.7%). The sections below report the retained items and their priorities within each domain.

***Learning Objectives**:* Experts converged on skill centric objectives aligned with communicative EFL design. The highest-ranked target was comprehensive skill development in listening, speaking, reading, writing, and translation (DV = 0.717; Agreement = 91.7%; IQR = 1.000; 𝑑 = 0.111), followed by learner autonomy and self-directed learning (DV = 0.700; Agreement = 91.7%; IQR = 1.000; 𝑑 = 0.117). Objectives integrating teamwork and collaboration also reached the parallel thresholds (DV = 0.672; Agreement = 91.7%; IQR = 1.000; 𝑑 = 0.149), as did grammar, vocabulary, culture knowledge and cross-cultural communication skills (both DV = 0.650; Agreement = 83.3%; IQR = 1.000; 𝑑 = 0.125). Creativity and innovation (DV = 0.633; Agreement = 83.3%; IQR = 1.000; 𝑑 = 0.111) and interest and motivation (DV = 0.633; Agreement = 83.3%; IQR = 1.000; 𝑑 = 0.139) were also retained.

By contrast, information and digital literacy (DV = 0.617; Agreement = 75.0%) and critical thinking (DV = 0.606; Agreement = 75.0%) were not accepted owing to dispersion (IQR = 1.25), indicating less alignment at module level for these broader competencies. Overall, the panel prioritized objectives directly related to language performance, whereas broader graduate attributes showed weaker consensus at the module level. Table 3 summarizes expert consensus on learning objectives.

**Table 3:** Expert consensus on learning objectives for the metaverse-supported blended EFL module.

Item	Learning Objectives	d	Agreement, %	IQR	m1	m2	m3	DV	Expert Consensus	Rank
1	Students will develop a clear understanding of English grammar rules, expand their English vocabulary as stipulated in the course book, and acquire insights into the culture, history, traditions, and values of English-speaking countries.	0.125	83.3%	1.000	0.450	0.650	0.850	0.650	ACCEPT	4
2	Students can improve language skills in listening, speaking, reading, writing, translation.	0.111	91.7%	1.000	0.517	0.717	0.917	0.717	ACCEPT	1
3	Students will develop cross-cultural communication skills in English.	0.125	83.3%	1.000	0.450	0.650	0.850	0.650	ACCEPT	5
4	Students are able to cultivate and apply creativity and innovative thinking in the context of English language usage, problem-solving, and communication.	0.111	83.3%	1.000	0.433	0.633	0.833	0.633	ACCEPT	6
5	Students are able to demonstrate proficiency in information literacy and digital literacy.	0.183	75.0%	1.250	0.417	0.617	0.817	0.617	REJECT	
6	Students are able to exhibit teamwork and collaboration skills.	0.149	91.7%	1.000	0.483	0.667	0.867	0.672	ACCEPT	3
7	Foster students’ learning interests and motivation to enhance their engagement and performance in English learning.	0.139	83.3%	1.000	0.433	0.633	0.833	0.633	ACCEPT	7
8	Students can cultivate and apply self-directed learning abilities within their English course.	0.117	91.7%	1.000	0.500	0.700	0.900	0.700	ACCEPT	2
9	Students are able to demonstrate critical thinking skills.	0.194	75%	1.250	0.417	0.600	0.800	0.606	REJECT	

*Note.* Acceptance was determined by three parallel criteria: Agreement (ratings of 4–5) ≥ 75%, IQR ≤ 1.0 (5-point scale), and fuzzy distance d ≤ 0.20. The defuzzified value (DV) is reported for priority ranking only. Triangular fuzzy mappings follow Table 2. The same criteria apply to Tables 4–8.

***Learning Content:*** Accepted content emphasized authentic materials that support situated communicative performance. Listening and speaking work and integrated reading and writing tasks shared the highest priority (both DV = 0.717; Agreement = 100.0% and 91.7%, respectively; IQR = 1.000; 𝑑 ≤ 0.111). Vocabulary and grammar (DV = 0.667; Agreement = 91.7%; IQR = 1.000; 𝑑 = 0.111), translation practice (DV = 0.656; Agreement = 91.7%; IQR = 1.000; 𝑑 = 0.144), and selected course book content (DV = 0.656; Agreement = 91.7%; IQR = 1.000; 𝑑 = 0.144) were also endorsed. ESP topics and English games designed to support language use both met all thresholds (DV = 0.606; Agreement = 83.3%; IQR = 1.000; d = 0.130). English literature and a generic virtual library were not retained due to insufficient agreement and dispersion. Overall, content items were accepted when their contribution to communicative performance was explicit. Table 4 presents the consensus results for learning content.

**Table 4:** Expert consensus on learning content for the metaverse-supported blended EFL module.

Item	Learning Content	d	Agreement, %	IQR	m1	m2	m3	DV	Expert Consensus	Rank
1	Reading and Writing Content	0.111	91.7%	1.000	0.517	0.717	0.917	0.717	ACCEPT	2
2	Vocabulary and Grammar	0.111	91.7%	1.000	0.467	0.667	0.867	0.667	ACCEPT	3
3	Listening and Speaking Content	0.097	100%	1.000	0.517	0.717	0.917	0.717	ACCEPT	1
4	Translation Practice	0.144	91.7%	1.000	0.467	0.650	0.850	0.656	ACCEPT	4
5	English Literature	0.178	75%	1.250	0.433	0.617	0.817	0.622	REJECT	
6	Content from the College English Course Book	0.144	91.7%	1.000	0.467	0.650	0.850	0.656	ACCEPT	5
7	ESP (English for Specific Purposes)	0.130	83.3%	1.000	0.417	0.600	0.800	0.606	ACCEPT	7
8	English Games	0.130	83.3%	1.000	0.417	0.600	0.800	0.606	ACCEPT	6
9	Virtual Library	0.215	66.7%	2.000	0.400	0.583	0.783	0.589	REJECT	

*Note*. Same acceptance and ranking criteria as Table 3.

***Instructional Strategies:*** The panel endorsed meaning focused pedagogical strategies that explicitly link immersion to communicative language use. The communicative approach ranked highest (DV = 0.750; Agreement = 91.7%; IQR = 0.000; d = 0.083), followed by immersive learning (DV = 0.717; Agreement = 91.7%; IQR = 1.000; d = 0.111) and contextual learning (DV = 0.700; Agreement = 91.7%; IQR = 1.000; d = 0.117). Task based language teaching and self-directed learning also met all thresholds. Gamified learning was retained when specified to support communicative practice. Content-based instruction (CBI), social network building, and emotional engagement were not accepted due to insufficient agreement or excessive dispersion. Table 5 summarizes expert evaluations of instructional strategies.

**Table 5:** Expert consensus on instructional strategies for the metaverse-supported blended EFL module.

Item	Instructional Strategies	d	Agreement, %	IQR	m_1_	m_2_	m_3_	DV	Expert Consensus	Rank
1	Immersive Learning	0.111	91.7%	1.000	0.517	0.717	0.917	0.717	ACCEPT	2
2	Task-based Language Learning (TBLT)	0.117	91.7%	1.000	0.483	0.683	0.883	0.683	ACCEPT	4
3	Content-based Instruction (CBI)	0.122	75%	1.250	0.417	0.617	0.817	0.617	REJECT	
4	Gamified Learning	0.136	83.3%	1.000	0.483	0.683	0.883	0.683	ACCEPT	5
5	Contextual Learning	0.117	91.7%	1.000	0.500	0.700	0.900	0.700	ACCEPT	3
6	Self-directed Learning	0.125	83.3%	1.000	0.450	0.650	0.850	0.650	ACCEPT	6
7	Communicative Approach	0.083	91.7%	0.000	0.550	0.750	0.950	0.750	ACCEPT	1
8	Social Network Building	0.150	75%	1.250	0.400	0.583	0.783	0.589	REJECT	
9	Emotional Engagement	0.199	58.3%	2.000	0.383	0.567	0.767	0.572	REJECT	

*Note*. Same acceptance and ranking criteria as Table 3.

***Learning Activities*:** Accepted activities emphasized authentic participation. Virtual cultural experiences emerged as the top activity (DV = 0.733; Agreement = 91.7%; IQR = 0.25; 𝑑 = 0.100), followed by role-play and speech, and lecture and discussion sequences designed for immediate practice (both DV = 0.717; Agreement = 91.7%; IQR = 1.000; 𝑑 = 0.111). Social interaction (DV = 0.667; Agreement = 91.7%; IQR = 1.000; 𝑑 = 0.111), virtual simulations (DV = 0.650; Agreement = 83.3%; IQR = 1.000; 𝑑 = 0.125), group learning tasks (DV = 0.650; Agreement = 83.3%; IQR = 1.000; 𝑑 = 0.125) and collaborative projects (DV = 0.617; Agreement = 83.3%; IQR = 1.000; 𝑑 = 0.122) were also accepted. A generic English learning games item, however, did not pass due to dispersion, indicating that gamified tasks require tighter pedagogical specification to command consensus. Table 6 summarizes consensus on the learning activities to include in the module.

**Table 6:** Expert consensus on learning activities for the metaverse-supported blended EFL module.

Item	Learning Activities	d	Agreement, %	IQR	m1	m2	m3	DV	Expert Consensus	Rank
1	Role-playing and speech Activities	0.111	91.7%	1.000	0.517	0.717	0.917	0.717	ACCEPT	2
2	Virtual Cultural Experience	0.100	91.7%	0.250	0.533	0.733	0.933	0.733	ACCEPT	1
3	Virtual Art Experience	0.125	75%	0.500	0.383	0.583	0.783	0.583	ACCEPT	8
4	Virtual Simulations	0.125	83.3%	1.000	0.450	0.650	0.850	0.650	ACCEPT	5
5	English Learning Games	0.133	75%	1.250	0.400	0.600	0.800	0.600	REJECT	
6	Lecture and Discussion	0.111	91.7%	1.000	0.517	0.717	0.917	0.717	ACCEPT	3
7	Group Learning Tasks	0.125	83.3%	1.000	0.450	0.650	0.850	0.650	ACCEPT	6
8	Social Interaction	0.111	91.7%	1.000	0.467	0.667	0.867	0.667	ACCEPT	4
9	Collaborative Projects	0.122	83.3%	1.000	0.417	0.617	0.817	0.617	ACCEPT	7

*Note*. Same acceptance and ranking criteria as Table 3.

***Assessment Methods***: Assessment preferences reflected a balanced approach combining summative and performance-oriented measures. Examination remained a core element (DV = 0.717; Agreement = 100.0%; IQR = 1.000; 𝑑 = 0.097), complemented by project presentation (DV = 0.683; Agreement = 83.3%; IQR = 1.000; 𝑑 = 0.136), assignment assessment (DV = 0.667; Agreement = 91.7%; IQR = 1.000; 𝑑 = 0.111), self-assessment and reflection (DV = 0.650; Agreement = 83.3%; IQR = 1.000; 𝑑 = 0.125) and participation records (DV = 0.617; Agreement = 83.3%; IQR = 1.000; 𝑑 = 0.122). Generic assessment scales and unspecific classroom observation were not retained. Table 7 summarizes expert consensus on assessment methods.

**Table 7:** Expert consensus on assessment for the metaverse-supported blended EFL module.

Item	Assessment	d	Agreement, %	IQR	m1	m2	m3	DV	Expert Consensus	Rank
1	Examination	0.097	100%	1.000	0.517	0.717	0.917	0.717	ACCEPT	1
2	Assignment Assessment	0.111	91.7%	1.000	0.467	0.667	0.867	0.667	ACCEPT	3
3	Course Participation Record	0.122	83.3%	1.000	0.417	0.617	0.817	0.617	ACCEPT	5
4	Learning Assessment Scale	0.178	66.7%	1.250	0.317	0.517	0.717	0.517	REJECT	
5	Project Presentation and Demonstration	0.136	83.3%	1.000	0.483	0.683	0.883	0.683	ACCEPT	2
6	Online Learning Record	0.122	75%	1.250	0.417	0.617	0.817	0.617	REJECT	
7	Self-Assessment and Reflection	0.125	83.3%	1.000	0.450	0.650	0.850	0.650	ACCEPT	4
8	Classroom Observation	0.189	66.7%	2.000	0.383	0.583	0.783	0.583	REJECT	

*Note*. Same acceptance and ranking criteria as Table 3.

***Learning Resources*:** Learning resources showed the most consistent rankings across experts. The metaverse learning space was unanimously prioritized (DV = 0.767; Agreement = 100.0%; IQR = 0.000; d = 0.056), followed by VR/AR videos (DV = 0.733; Agreement = 100.0%; IQR = 1.000; d = 0.089) and instructional videos with e slides (both DV = 0.700; Agreement = 91.7%; IQR = 1.000; d = 0.117). MOOCs (DV = 0.650; Agreement = 83.3%; IQR = 1.000; d = 0.125) and dictionary and grammar tools (DV = 0.633; Agreement = 83.3%; IQR = 1.000; d = 0.111) were also accepted. Learning cards, academic books, and literary works were not retained due to insufficient agreement or dispersion. Table 8 reports the consensus results for learning resources.

**Table 8:** Expert consensus on learning resources for the metaverse-supported blended EFL module.

Item	Learning Resources	d	Agreement, %	IQR	m1	m2	m3	DV	Expert Consensus	Rank
1	MOOC Courses	0.125	83.3%	1.000	0.450	0.650	0.850	0.650	ACCEPT	5
2	Instructional Videos	0.117	91.7%	1.000	0.500	0.700	0.900	0.700	ACCEPT	3
3	Electronic Textbooks and Slides (PPT)	0.117	91.7%	1.000	0.500	0.700	0.900	0.700	ACCEPT	4
4	Virtual Learning Space (Metaverse learning space)	0.056	100%	0.000	0.567	0.767	0.967	0.767	ACCEPT	1
5	VR/AR Videos	0.089	100%	1.000	0.533	0.733	0.933	0.733	ACCEPT	2
6	Learning Cards	0.192	58.3%	2.000	0.350	0.550	0.750	0.550	REJECT	
7	Dictionary and Grammar Tools	0.111	83.3%	1.000	0.433	0.633	0.833	0.633	ACCEPT	6
8	Academic Books	0.133	66.7%	2.000	0.400	0.600	0.800	0.600	REJECT	
9	English Literary Works	0.144	66.7%	1.250	0.367	0.567	0.767	0.567	REJECT	

*Note*. Same acceptance and ranking criteria as Table 3.

Taken together, the accepted and ranked items constitute a consolidated blueprint for a metaverse supported blended EFL module. At the objective level, the blueprint prioritizes comprehensive language skill development and learner autonomy. Instructional strategies emphasize communicative, immersive, contextual, and task-based approaches. Learning activities focus on virtual cultural experiences, role-play, brief lecture plus discussion, social interaction, simulations, and collaborative tasks. Content centers on listening and speaking, reading and writing, vocabulary and grammar, translation practice, and selected course book materials. Assessment combines examinations with project demonstrations, assignments, self-assessment and reflection, and participation records. Learning resources are predominantly digital and immersive, with the metaverse learning space and VR/AR media receiving the highest priority.

***Panel level concordance and ranking stability.*** As a robustness check, resampling based checks indicated that the within-domain DV rankings were highly stable under LOO analyses (mean Spearman’s ρ = 0.967; range 0.890–0.989) and moderately stable under bootstrap resampling (mean ρ = 0.779; 95% interval 0.533–0.925; B = 5,000), suggesting that the prioritization is not driven by any single expert and remains reasonably robust under repeated resampling. For descriptive context, Kendall’s coefficient of concordance indicated limited overall ranking agreement across heterogeneous items (*W* = 0.108, df = 53, p = 0.071). These statistics characterize ranking stability and coordination, while item inclusion remains determined by the pre-specified thresholds (Agreement, IQR, *d*).

**Fig 1:**
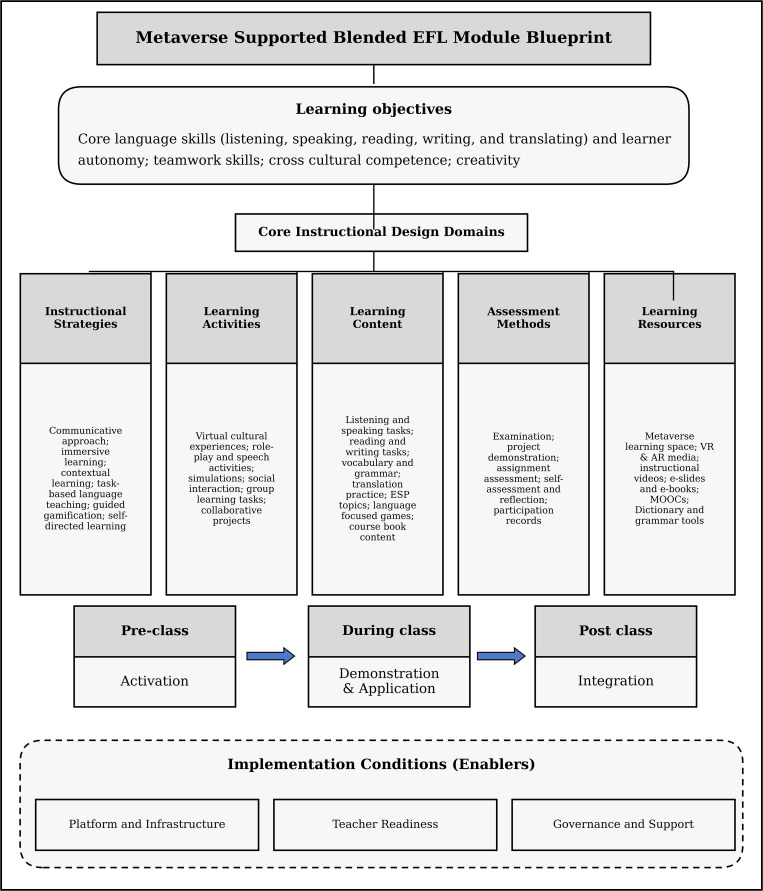
Integrated blueprint for a metaverse-supported blended EFL module.

Fig 1 integrates the expert-validated design elements across learning objectives, instructional strategies, learning activities, learning content, assessment methods, and learning resources into a coherent module blueprint. The elements are organized within a blended learning structure (pre-class, in-class, and post-class), while implementation conditions are shown as enabling factors rather than additional consensus outcomes.

## 5. Discussion

This study used FDM to distil and prioritize design elements for a metaverse supported blended EFL module in undergraduate education. Across domains, retained items align with constructive alignment by linking intended outcomes, learning activities, and assessment [2,3]. Experts endorsed core language outcomes, communicative and task centred pedagogies, authentic interactional activities, balanced assessment, and a digital immersive resource set. Overall, the metaverse was valued when explicitly tied to communicative language use rather than technological novelty [5]. The study contributes an expert consensus ranked blueprint, not evidence of post implementation effectiveness.

At the pedagogical level, the highest priority strategies (communicative, immersive, contextual, and task-based instruction) map onto established EFL pedagogy [7–9]. Immersion and presence alone were not viewed as sufficient without meaning focused language use [6]. Gamified learning was endorsed conditionally, only when it supported communicative practice, consistent with the view that game elements should be designed to support learning and aligned with tasks and feedback [10]. Accordingly, gamification is best treated as a wrapper around communicative tasks with explicit performance evidence.

For activities, experts prioritized virtual cultural experiences, role play, brief lecture plus discussion for immediate practice, social interaction, simulations, and collaborative tasks. These selections favour authentic participation and mirror task-based frameworks in which learners negotiate meaning and co construct output [7,8]. Lower support for loosely specified activities suggests that each activity should state its communicative intent and evidence requirements. For curriculum teams, a practical specification is to define each activity by target language function(s), interaction pattern, expected artefact(s), and assessment evidence.

Regarding content, the panel endorsed listening–speaking, reading–writing, vocabulary–grammar, and translation practice, alongside curated course book materials and language focused ESP and games. Items were retained when they clearly supported situated communicative performance; generic repositories, for example, an unspecified “virtual library”, were not. This reflects an alignment logic in which content is selected to enable targeted performance in immersive tasks rather than broad coverage [2,3]. Broader graduate attributes received lower consensus, suggesting a preference for outcomes proximal to language performance.

Assessment preferences indicated a balanced set: examinations alongside project demonstrations, assignments, self-assessment and reflection, and participation records. This combination supports summative accountability and formative regulation [35,36]. The rejection of generic scales and observation notes points to evidence of performance stance: assessment should be derived from aligned artefacts (e.g., recorded interactions, role-play outputs, demonstrations) and process indicators (e.g., participation logs, reflective entries), with criteria made explicit to support transparency and fairness.

Resources showed the clearest coordination: the metaverse learning space and VR/AR media were highly prioritized, complemented by instructional videos, e-slides, MOOCs, and dictionary and grammar tools. This configuration is consistent with evidence that immersive media can support experiential and contextual learning when goals, scaffolding, and feedback are explicit [6,37]. Implementation therefore requires orchestration and a teacher readiness plan, for example, task scripting, facilitation, troubleshooting, and assessment literacy, particularly where technical support is limited.

To operationalize implementation, we propose a Teacher Readiness Framework that synthesizes key readiness domains, observable indicators, and suggested supports aligned with the ranked blueprint. The framework is intended as an implementation heuristic to guide professional development and staged adoption. The framework is provided in S1 Table (Supplementary material).

Equity, ethics, scalability, and sustainability are necessary implementation considerations in metaverse supported EFL. Equity involves device and bandwidth variability, accessibility accommodations, and the risk that high-immersion designs may disadvantage learners facing digital poverty. Governance and ethics require informed consent, data minimization, privacy protection, and clear policies for storing and using interaction traces. Scalability and sustainability depend on recurring costs (hardware, software, maintenance, training time), staffing capacity, and platform and content longevity. These constraints support staged adoption, beginning with lower barrier configurations and progressing toward richer immersion as infrastructure and teacher readiness mature, while aligning innovation with broader quality and equity agendas, such as SDG 4 and SDG 10.

Methodologically, item inclusion was determined by the prespecified parallel thresholds (Agreement ≥ 75%, IQR ≤ 1.0, d ≤ 0.20), and retained items were prioritized by DV within each domain. As reported in the Findings, resampling based stability checks (LOO and bootstrap Spearman’s ρ) supported the robustness of the DV induced priority ordering under perturbations in panel composition. Kendall’s *W* was reported as a supplementary descriptive summary of overall coordination across heterogeneous items. Accordingly, the ranked blueprint provides a reproducible basis for subsequent validation and comparative evaluation.

Limitations include the panel size (n = 12) and its grounding in Chinese higher education, which may limit transferability. The findings should be interpreted as threshold-based expert consensus design specifications rather than effectiveness evidence. Future work should evaluate the blueprint in live courses, examine learning gains against aligned outcomes, and incorporate teacher and student perspectives on usability and equity. To strengthen cross-context validity, future work should also broaden expert representation to include international EFL and metaverse specialists and/or conduct an independent external audit of the finalized blueprint. Future implementation research should also develop and validate a staged adoption (maturity) model for metaverse-supported EFL based on multi-site evidence and stakeholder feedback. Cross-institutional and cross-national comparisons can clarify which elements are robust versus context-sensitive. As metaverse ecosystems evolve, controlled evaluations of integrations with AI (e.g., adaptive feedback or conversational agents) are warranted to test additive value beyond the present blueprint.

## 6. Conclusion

This study provides a consensus based, ranked blueprint for designing a metaverse supported blended EFL module in undergraduate education. The blueprint operationalizes constructive alignment by specifying prioritized objectives, strategies, activities, assessment, and resources for immersive delivery. Core priorities include overall EFL proficiency development and learner autonomy, communicative and task-based instruction, authentic activities such as cultural experiences, role play, simulations, and collaborative tasks, and assessment that combines summative measures with performance based and reflective evidence. Resources prioritize the metaverse learning space, VR and AR media, and structured digital supports. Although overall ranking concordance was modest, the strict item level thresholds ensure that retained elements reflect convergent expert endorsement and are suitable for immediate course design and staged adoption.

These conclusions are limited to expert consensus design specifications and do not imply post implementation effectiveness. Future work should evaluate the blueprint in live courses and examine learning outcomes alongside teacher and learner experiences. Implementation should be adapted to local constraints, for example, device and bandwidth variability, accessibility needs, and data governance requirements, through staged adoption.

## Supporting information

S1 Table Teacher Readiness Framework for implementing the metaverse-supported blended EFL module. This table summarizes readiness domains, observable indicators, and suggested supports for staged adoption.
(DOCX)
