# Supplementary material for: Expert consensus on designing a metaverse supported blended EFL module in chinese higher education: A Fuzzy Delphi method
Source: PLoS One. 2026 Apr 16;21(4):e0347027. doi: 10.1371/journal.pone.0347027 (PMC13086304; doi:10.1371/journal.pone.0347027)
Supplement: S1 Table — This table summarizes readiness domains, observable indicators, and suggested supports for staged adoption. (DOCX) [file pone.0347027.s001.docx]

Table S1. Teacher readiness framework for staged adoption of a metaverse supported EFL module

| Readiness domain | Observable indicators | Suggested supports |
| --- | --- | --- |
| 1. Constructive alignment & task design | Documented Outcomes→Activities→Assessment map; tasks specify language function(s), interaction pattern/roles, artefact(s), and evidence | Alignment templates; task-script library; exemplar rubrics + samples; peer review of task plans |
| 2. Platform competence & troubleshooting | Sets up spaces/sessions and permissions; resolves common failures; defined low-bandwidth/device-light fallback (2D/async) | Pre-flight checklist; minimum tech specs; helpdesk/TA support; contingency pack (2D + async) |
| 3. Facilitation & orchestration (immersive) | Structured interaction (grouping/rotation/roles/timeboxing); debrief links performance to outcomes; manages cognitive load | Facilitation prompt bank; collaboration templates; debrief question bank; interaction norms |
| 4. Assessment literacy & performance evidence | Uses aligned artefacts (recordings/role-plays/demos) + process evidence (participation/reflection); explicit criteria; feedback cycle | Assessment toolkit (rubrics/logs); rater calibration; feedback exemplars and guidance |
| 5. Privacy/ethics & governance | Consent in place; data minimization/access control; policy for trace use/retention; incident escalation route | Institutional policy summary; consent scripts; privacy checklist; secure storage/access governance |

Note. This framework is proposed as an implementation heuristic to guide professional development and staged adoption.
